# Crosstalk between Yeast Cell Plasma Membrane Ergosterol Content and Cell Wall Stiffness under Acetic Acid Stress Involving Pdr18

**DOI:** 10.3390/jof8020103

**Published:** 2022-01-21

**Authors:** Ricardo A. Ribeiro, Cláudia P. Godinho, Miguel V. Vitorino, Tiago T. Robalo, Fábio Fernandes, Mário S. Rodrigues, Isabel Sá-Correia

**Affiliations:** 1 iBB—Institute for Bioengineering and Biosciences, Instituto Superior Técnico, Universidade de Lisboa, Av. Rovisco Pais, 1049-001 Lisbon, Portugal; ricardo.ribeiro@tecnico.ulisboa.pt (R.A.R.); claudia.godinho@tecnico.ulisboa.pt (C.P.G.); fernandesf@tecnico.ulisboa.pt (F.F.); 2 Associate Laboratory i4HB—Institute for Health and Bioeconomy at Instituto Superior Técnico, Universidade de Lisboa, Av. Rovisco Pais, 1049-001 Lisbon, Portugal; 3 Department of Bioengineering, Instituto Superior Técnico, Universidade de Lisboa, Av. Rovisco Pais, 1049-001 Lisbon, Portugal; 4 BioISI—Biosystems and Integrative Sciences Institute, Faculdade de Ciências, Universidade de Lisboa, Campo Grande 16, 1749-016 Lisbon, Portugal; mvvitorino@fc.ul.pt (M.V.V.); ntrobalo@fc.ul.pt (T.T.R.); mmrodrigues@fc.ul.pt (M.S.R.); 5 Departament of Physics, Faculdade de Ciências, Universidade de Lisboa, Campo Grande 16, 1749-016 Lisbon, Portugal

**Keywords:** *Saccharomyces cerevisiae*, adaptation and tolerance to acetic acid, cell envelope, plasma membrane, cell wall stiffness, cell wall remodeling, lipid homeostasis, ergosterol, ABC transporters, Pdr18, intracellular pH

## Abstract

Acetic acid is a major inhibitory compound in several industrial bioprocesses, in particular in lignocellulosic yeast biorefineries. Cell envelope remodeling, involving cell wall and plasma membrane composition, structure and function, is among the mechanisms behind yeast adaptation and tolerance to stress. Pdr18 is a plasma membrane ABC transporter of the pleiotropic drug resistance family and a reported determinant of acetic acid tolerance mediating ergosterol transport. This study provides evidence for the impact of Pdr18 expression in yeast cell wall during adaptation to acetic acid stress. The time-course of acetic-acid-induced transcriptional activation of cell wall biosynthetic genes (*FKS1*, *BGL2*, *CHS3*, *GAS1*) and of increased cell wall stiffness and cell wall polysaccharide content in cells with the *PDR18* deleted, compared to parental cells, is reported. Despite the robust and more intense adaptive response of the *pdr18*Δ population, the stress-induced increase of cell wall resistance to lyticase activity was below parental strain levels, and the duration of the period required for intracellular pH recovery from acidification and growth resumption was higher in the less tolerant *pdr18*Δ population. The ergosterol content, critical for plasma membrane stabilization, suffered a drastic reduction in the first hour of cultivation under acetic acid stress, especially in *pdr18*Δ cells. Results revealed a crosstalk between plasma membrane ergosterol content and cell wall biophysical properties, suggesting a coordinated response to counteract the deleterious effects of acetic acid.

## 1. Introduction

Acetic acid is a major inhibitory compound in industrial bioprocesses, in particular in lignocellulosic biorefineries. For this reason, a better understanding of the mechanisms underlying yeast adaptation and tolerance to this stress is essential for the rational improvement of yeast cell robustness [1,2,3,4]. At a pH below acetic acid p*K*_a_ (4.75 at 25 °C) [5], the undissociated form of the acid (CH_3_COOH) is able to cross the plasma membrane lipid bilayer [1,2,6]. Once inside the cell interior and in the neutral cytosol, acetic acid dissociates, leading to the release of protons (H^+^) and the consequent intracellular acidification, and accumulation of the counter-ion (CH_3_COO^−^). Ultimately, acetic acid dissociation causes the inhibition of growth kinetics and metabolism as the result of multiple toxicity mechanisms [1,2].

Several genome-wide approaches have allowed a more thorough understanding of the mechanisms involved in yeast’s response and adaptation to acetic acid stress [2,7,8,9,10,11,12,13,14,15,16]. Among these mechanisms is the alteration of the molecular composition and biophysical properties of the cell envelope, acting as the first barrier of protection against challenging environmental conditions [2,17,18,19,20,21,22,23]. The cell wall is a layered structure, with an inner layer mainly composed of β-glucans and chitin, and an outer layer constituted by highly glycosylated cell wall mannoproteins (CWP). The biochemical composition and the organization of the cell wall are dynamic and have been reported to change during adaption to different environmental conditions, impacting cell endurance and survival [20,21,24,25,26]. Changes in cell wall biophysical properties, such as the stiffness of the cell surface, mostly dependent on the cross-linking between β-glucans and chitin [24], have been reported to occur in response to industrially relevant stresses [20,25,26]. The time-course of the alterations occurring at the level of the cell wall during adaptation of a yeast cell population to sudden exposure to a sub-lethal stress induced by acetic acid was recently reported. The induced increase of cell wall resistance to lyticase activity, stiffness and content in β-glucans was described [20]. In addition, different studies have shown that several genes coding for proteins required for cell wall polysaccharide synthesis and regulation are transcriptionally responsive to acetic acid stress and/or determinants of acetic acid stress tolerance [7,9,10,12,14,20]. These responses guarantee a robust adaptive response, which is essential to limit the futile cycle associated with the re-entry of the acid form after the active expulsion of acetate from the cell interior [20].

Plasma membrane remodeling is also essential for acetic acid tolerance in yeast. The adequate incorporation of sphingolipids and ergosterol in the plasma membrane was found to decrease plasma membrane non-specific permeabilization under acetic acid stress, both in *Saccharomyces cerevisiae* and in the highly tolerant food spoilage yeast species *Zygosaccharomyces bailii* [17,18,19,27]. The content of ergosterol is critical for plasma membrane stabilization and adequate selective permeability, limiting the passive diffusion of lipophilic toxic compounds into the cell [28,29,30]. Ergosterol is also essential for the formation of lipid-raft microdomains that modulate the activity of membrane-embedded proteins [28,30]. Lipid raft microdomains are also involved in the proper localization and trafficking of several mannoproteins, in particular glycosyl-phosphatidylinositol (GPI)-anchored proteins, associated with cell wall integrity [31,32,33,34,35]. Cell wall polysaccharides are synthetized at the plasma membrane level, and some proteins involved in cell wall integrity sensing or cell wall remodeling exhibit domains that interact with the plasma membrane [22,36,37,38,39], reinforcing the importance of plasma membrane homeostasis. The plasma membrane is the home for multidrug/multixenobiotic resistance (MDR/MXR) transporters of the ATP-binding cassette (ABC) Superfamily and of the Major Facilitator Superfamily (MFS), required for yeast resistance to multiple stresses [40,41,42]. These transporters are considered to contribute to a decrease in the intracellular concentration of specific toxic compounds, either by actively pumping them out of the cell and/or by promoting physical or chemical changes in the plasma membrane, thus affecting their partition. The MFS transporters Tpo2, Tpo3 and Aqr1 have been implicated in the active expulsion of acetate from the cell interior, thus reducing the deleterious effects of acetic acid counterion accumulation [41]. However, the expression, functioning and recycling of these and other plasma membrane transporters is energetically expensive to the cell [43,44]. Yeast cell adaptation to acetic acid stress also involves the remodeling of plasma membrane composition and organization to restrict the diffusional entry of the liposoluble acid form into the cell. An identified player in this process is the plasma membrane ABC transporter Pdr18 [17]. The expression of this pleiotropic drug resistance (PDR) transporter confers resistance to a wide range of chemical and physical stresses, including acetic acid stress [17,45,46,47,48]. Pdr18 was proposed to mediate ergosterol transport at the plasma membrane, maintaining adequate plasma membrane physical properties under stress induced by acetic acid, which was found to lead to the reduction of ergosterol content [17]. Through its action, Pdr18 allows the maintenance of ergosterol content and plasma membrane order in cells growing exponentially in the presence of acetic acid [17]. The transcriptional up-regulation of *PDR18* observed during the latency period induced by acetic acid stress was found to be essential to counteract the induced increase of plasma membrane non-specific permeability and dissipation of transmembrane electrochemical potential [17]. Overall, Pdr18 is considered essential for adaptation and tolerance to acetic acid stress by promoting the adequate essential physiological function of the yeast plasma membrane [17,47].

In the present work, the influence of the expression of *PDR18* in yeast cell wall properties at the molecular and biophysical levels, under acetic acid-induced stress, was examined. The time-course effect of *PDR18* expression in counteracting the decrease of ergosterol concentration and of intracellular pH, and in cell wall resistance to lyticase activity, cell wall stiffness (by atomic force microscopy-AFM), and cell wall polysaccharide composition (based on fluorescence microscopy analysis) were examined. Collectively, the results obtained provide evidence for the crosstalk between the yeast plasma membrane ergosterol content, involving Pdr18 activity, and cell wall biophysical properties, suggesting a coordinated and robust yeast response to counteract the deleterious effects of acetic acid stress. 

## 2. Materials and Methods

### 2.1. Yeast Strains and Growth Conditions

The parental strain *Saccharomyces cerevisiae* BY4741 (*MATa, his3*Δ*1, leu2*Δ*10, met15*Δ*0, ura3*Δ*0*) and the derived deletion mutant *pdr18*Δ strain, obtained from the EUROSCARF collection, were used in this study. The strains were maintained at −80 °C in YPD media supplemented with 30% (*v*/*v*) glycerol. Cells were cultivated at 30 °C, with orbital agitation (250 rpm), in minimal growth medium supplemented with amino acids and uracil (MM4). MM4 contains 1.7 g/L yeast nitrogen base without amino acids and ammonium sulfate (Difco, Michigan, MI, USA), 20 g/L glucose (Merck, Darmstadt, Germany), 2.65 g/L (NH_4_)_2_SO_4_ (ITW reagents, Panreac AppliChem, Chicago, IL, USA), 20 mg/L methionine, 20 mg/L histidine, 60 mg/L leucine and 20 mg/L uracil (all from Sigma, St. Louis, MO, USA). The medium pH was adjusted to 4.0 with HCl. When stated, MM4 medium was supplemented with 60 mM acetic acid (Fluka, Buchs, Switzerland.) using a solution of 5 M acetic acid, set to pH 4.0 with NaOH. For all the growth experiments, MM4, supplemented or not with 60 mM acetic acid, was inoculated to an initial optical density at 600_nm_ (OD_600nm_) of 0.1 ± 0.05 using mid-exponential cells harvested by filtration (Whatman, Maidstone, UK) from cultivation in fresh MM4 medium (pH 4.0) without acetic acid supplementation. Growth was followed by measuring culture OD_600nm_.

### 2.2. Ergosterol Quantification

At adequate time-points during parental and *pdr18*Δ strain cultivation in either MM4 or MM4 supplemented with 60 mM acetic acid (pH 4.0), cells corresponding to 100 OD_600nm_ units were harvested (7000× *g*, 5 min) for ergosterol quantification. Total cellular ergosterol was quantified based on a spectrophotometric method [49]. The absorbance of the sterol-containing layer was traced between 200 and 300 nm to confirm the success of the extraction by visualization of a characteristic four-peaked curve [50]. Optical density at 281.5 and 230 nm was used to determine the ergosterol content using equations previously reported, normalized to the wet weight of the sample [50]. Results were obtained from two independent biological replicates, each arising from three quantification replicates. Results were obtained using at least three independent experiments, and statistically significant differences were identified using one-way analysis of variance (ANOVA).

### 2.3. Determination of Intracellular pH (pHi) by Flow Cytometry

For the determination of the intracellular pH values (pHi), both parental and *pdr18*Δ strains were cultivated in either MM4 or MM4 supplemented with 60 mM acetic acid (pH 4.0). At adequate time-points, 0.5 OD_600nm_ units were harvested by filtration using a pore size of 0.2 μm (Whatman, Maidstone, UK) and eluted in 250 µL of citrate-phosphate buffer, to which was added SNARF-4F-5-(and-6)-carboxylic acid, acetoxymethyl ester and acetate (Invitrogen, Waltham, MA, USA), at a final concentration of 20 µM. After an incubation period of 20 min (at 30 °C with 250 rpm orbital agitation), cells were washed, resuspended in 250 µL of citrate-phosphate buffer pH 3.0 without the probe, and analyzed immediately. Flow cytometric analysis was performed on a BD Accuri C6 Plus flow cytometer (Becton, Dickinson and Company, Franklin Lakes, NJ, USA). The probe fluorescence emission was collected via FL2 585/40 and FL4 675/25 filters. All experiments were repeated using at least two independent biological replicates. A fixed total of 40,000 events per sample were acquired using a slow flow rate (14 µL/min). The FL2/FL4 ratio of fluorescence emission was calculated for every population. 

The fluorescence emission collected in both channels from non-labeled exponentially growing parental and *pdr18*Δ cell suspensions was also measured to subtract the background cell fluorescence from each channel emission signal. The determination of intracellular pH was assessed by fitting the ratios of fluorescence in the equations obtained from calibration curves for parental and *pdr18*Δ populations, which converted the fluorescence ratio to pHi values. For this, the cells were incubated in 250 µL of citrate-phosphate buffer supplemented with SNARF-4F-5-(and-6)-carboxylic acid, acetoxymethyl ester, and acetate at a defined pH from 5.5 to 8.0 for 1 h at 30 °C. The calibration curves (Supplementary Figure S1) were obtained by plotting the fluorescence ratio of the different samples as a function of the pH of the buffer in which they were incubated and fitted with a second-order polynomial function.

### 2.4. Yeast Cell Wall Susceptibility to Lyticase

To monitor yeast cell wall structural alterations during cultivation in MM4 supplemented or not with 60 mM acetic acid, a lyticase susceptibility assay was conducted as described before [51]. The lyticase used (β-1,3-glucanase from *Arthrobacter luteus*; Sigma; batch 0000109539) contained β-(1→3)-glucan laminaripentaohydrolase, β-(1→3)-glucanase, protease, and mannanase activities. Briefly, cells of the parental and *pdr18*Δ strains were cultivated in MM4 liquid medium, either supplemented or not with 60 mM acetic acid, at pH 4.0, and were harvested by filtration at adequate time-points during cultivation. Cells were washed with bidistilled and deionized water and used to inoculate 100 mL Erlenmeyer flasks containing 50 mL of 0.1 mM phosphate buffer (pH 6.6), to a final OD_600nm_ of 0.5. After the addition of 18,000 U/mL of lyticase, cell lysis was followed by measuring the decrease of OD_600nm_ for each cell suspension. Values were converted to the percentage of the initial OD_600nm_ value. Cell susceptibility to lyticase is represented as the maximum specific lysis rate, defined as the absolute value of the slope of the trend line that best fits the linear part of the semi-logarithmic plot of the lysis curve. Results arise from at least two independent experiments, and statistically significant differences were identified using one-way ANOVA.

### 2.5. Measurement of the Young’s Modulus of the Cell Surface by Atomic Force Microscopy (AFM)

For the determination of the cell wall Young’s modulus, which is the quantitative expression of the cell surface elasticity that reflects the stiffness, cells of the *pdr18*Δ strain were grown and harvested at adequate time-points during cultivation, as described above for the quantification of the ergosterol content, pHi, and susceptibility to lyticase assays. The immobilization of the *pdr18*Δ cells for AFM analysis and the Force-Distance measurements for determination of the apparent Young’s modulus was performed as previously described [20]. Briefly, cell suspensions of OD_600nm_ of 0.2 in 5 mL of bidistilled deionized water were prepared and filtered through a polycarbonate membrane (Whatman) with a pore size of 3 μm, close to the yeast cell longitudinal size. The filters were washed once with 15 mL of bidistilled deionized water. AFM images and Force-Distance measurements were analyzed with a PicoLE Molecular Imaging system recorded in contact mode, using a microlever MSCT-F (Bruker, Billerica, MA, USA) with a nominal stiffness of 0.6 N/m and nominal tip radius of 10 nm in all experiments. Force spectroscopy mapping, consisting of 32 × 32 approach/retract force-displacement (FD) curves, was performed on the cell surface. The maximum deflection of the AFM cantilever was set constant, yielding a maximum applied force of ≈ 30 nN, and the tip-sample approach velocity was about 0.5 μm/s. An area in the cell was selected so that bud scars were excluded and an approximate total of 100 approach/retract force-displacement curves were obtained, from which the median value was kept. A total of 32 cantilevers were used, and the spring constant of the cantilevers was calibrated using the Sader method [52]. Each cantilever was used to measure cells at numerous time-points, and the order in which cells of different conditions were measured was randomized to reduce bias. However, it was not possible to use the same cantilever for all the conditions tested. The force-distance grids were analyzed for the determination of the apparent Young’s modulus, obtained by adjusting the Derjaguin-Muller-Toporov (DMT) contact model to the approach curves. The force-distance grids were processed by in-house software, developed using Wolfram Mathematica (Wolfram Research, Champaign, IL, USA). The results were obtained from at least three independent experiments and from the analysis of at least 14 cells for each condition. Statistical significance of differences among conditions was evaluated by applying the one-tailed Mann–Whitney U test.

### 2.6. Transcriptional Analysis of Cell Wall Biosynthetic and Regulatory Genes

For the transcriptional analysis of cell wall biosynthetic and regulatory genes, cells from the *pdr18*Δ deletion mutant strain were harvested during cultivation as described above for other assays. Exponentially growing cells from the parental strain cultivated in unstressed conditions were also harvested, to be used as a reference value in transcript quantification. Total RNA extraction was performed by the hot phenol method [53]. The quantitative real-time Reverse Transcription–PCR (qRT–PCR) protocol was used to determine the mRNA levels from *RLM1*, *FKS1*, *FKS2*, *BGL2*, *CHS3*, *CRH1*, *GAS1,* and *PRM5* genes following the manufacturer’s instructions. Primers were designed using Primer Express software V3.0 (Applied Biosystems) and are listed in Supplementary Table S1. The RT–PCR reaction was conducted in a thermal cycler block (Cleaver GTC965), and the qPCR was conducted in QuantStudio 5 (Applied Biosystems) using NZYSpeedy qPCR Green Master Mix (NZYTech). The *ACT1* mRNA level was used as the internal control. The value obtained for each target gene at the initial time-point (0 h) for the parental strain cells cultivated under unstressed conditions was set as 1, and the remaining values are relative to this reference value. The results were obtained from at least three independent experiments, and statistically significant differences were tested using one-way ANOVA.

### 2.7. Assessment of Cell Wall Polysaccharide Content by Fluorescence Microscopy

The staining methodology to assess cell wall main polysaccharide content was adapted from that described by Pradhan et al. [54] and as described before [20]. Fc-Dectin 1-Alexa 633, Calcofluor White, and Concanavalin A-FITC were used as staining compounds that bind glucans, chitin, and mannans, respectively. Cells of *pdr18*Δ strain population were harvested during cultivation, as described above. Cell suspensions of OD_600nm_ of 0.5 were incubated with 0.75 µg/mL Fc-Dectin 1 (Sino Biological, Beijing, China) in FACS buffer for 45 min on ice. After centrifugation at 4300× *g* for 5 min at 4 °C, the pellet was washed with 200 µL FACS buffer. Then, cells were incubated for 30 min with 1:200 anti-human IgG conjugated with Alexa Fluor 633 (ThermoFisher Scientific, Waltham, MA, USA), 50 µg/mL Calcofluor White (Fluka), and 50 µg/mL Concanavalin A conjugated with Fluorescein (Sigma). After centrifugation at 4300× *g* for 5 min at 4 °C, the pellet was washed with 200 µL FACS buffer and resuspended in 100 µL FACS buffer. These cell suspensions were immobilized in 2.2% agarose in PBS 1× mounted on a gene frame of 1.0 × 1.0 cm (ThermoFisher Scientific, Waltham, MA, USA). All samples were examined using a Leica TCS SP5 (Leica Microsystems CMS GmbH, Mannheim, Germany) inverted confocal microscope (DMI6000). A 63× apochromatic water immersion objective with a numerical aperture (NA) of 1.2 (Zeiss, Jena, Germany) was used for all experiments. Confocal microscopy was used for the FC-Dectin1-Alexa 633 channel, and excitation was carried out through a He–Ne laser line at 633 nm. Fluorescence emission was collected in this channel between 640 and 770 nm. For the Calcofluor White channel, 2-photon excitation microscopy measurements were carried out with a Spectra-Physics Mai Tai BB laser set for excitation at 780 nm. Calcofluor white emission was retrieved between 390 and 480 nm. Confocal microscopy was employed for the Concanavalin A-FITC channel, and excitation set at 488 nm was carried out using an Argon laser. Emission was collected between 495 and 580 nm. The fluorescence microscopy images for data analysis were collected at 2048 × 2048 resolution and processed using ImageJ. The regions of interest (ROI) corresponding to the cellular surface were defined, and average pixel fluorescence intensities within these ROIs were determined for each channel. The average background fluorescence was subtracted to obtain the calculated values. For each channel, the median of the intensity values obtained for *pdr18*Δ cells at the initial time-point (0 h) was set as 1, and the remaining values were relative to this reference value.

The triple staining of the cells was carried out to guarantee that the analysis of β-glucans, chitin, and mannans referred to the same cell population. The results of the median intensity fluorescence were obtained from the analysis of at least 33 cells from two independent cultivation experiments. The statistical significance of differences among conditions was evaluated by applying the one-tailed Mann–Whitney U test.

## 3. Results

### 3.1. Expression of PDR18 Is Required to Counteract the Decrease of Ergosterol Content and to Reduce the Time for Intracellular pH (pHi) Recovery following Acidification Induced by Acetic Acid Stress

**Figure 1:**
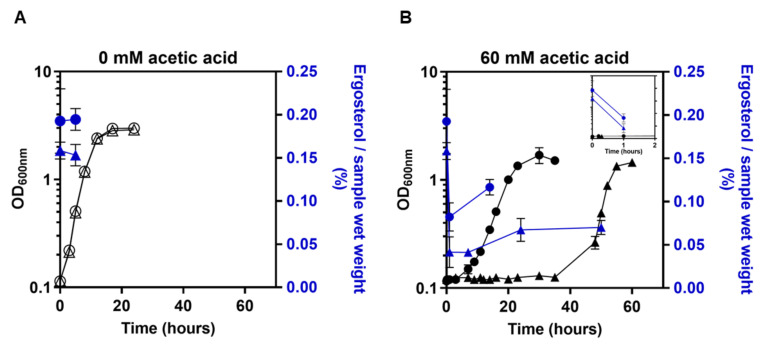
Alteration of the cellular ergosterol content of populations of the parental strain, *S. cerevisiae* BY4741 (circles), and of this strain with the *PDR18* gene deleted (triangles) during cultivation in the absence or presence of acetic acid stress. Cells of the parental and *pdr18*Δ strains were harvested during cultivation in MM4 (**A**) or MM4 supplemented with 60 mM acetic acid (**B**). A zoomed view of the first 2 h of cultivation in the presence of the acid is shown in (**B**), for which the left and right axis were maintained. Values are the means of at least two independent experiments, and error bars indicate standard deviations.

The deletion of *PDR18* leads to a more extended period of latency in the presence of 60 mM of acetic acid at pH 4.0, compared with the parental strain (50 h versus 13 h) (Figure 1), consistent with a former observation [17]. In the previous article, Pdr18 was found to be essential for the maintenance of the plasma membrane ergosterol content in unstressed cells and adapted cells exponentially growing in the presence of acetic acid [17]. In the present work, cellular ergosterol was also assessed during the latency phase induced by acetic acid, in cells expressing *PDR18* or not (Figure 1). Cells expressing *PDR18* exhibit a higher ergosterol content than *pdr18*Δ cells when cultivated in unstressed conditions (*p* < 0.05; one-way-ANOVA) (Figure 1A). The results demonstrate, for the first time, that the exposure of yeast cells to the referred acetic acid stress leads to a dramatic reduction in the ergosterol content after only 1 h of cultivation (decrease from 0.2% to 0.09% of the cell’s wet weight for the parental strain and from 0.15% to 0.04% of the cell’s wet weight for the *pdr18*Δ strain) (Figure 1B). This reduction was found to be statistically significant (*p* < 0.05; one-way ANOVA). The average levels of ergosterol in *pdr18*Δ cells after 7 h of cultivation in the presence of acetic acid were still low (0.04% of the cell’s wet weight). However, after 24 h of cultivation, these levels increased to 0.06% of the cell’s wet weight, this value being close to the value quantified after 50 h of cultivation, during the exponential growth phase (Figure 1B) and the values obtained before for acetic acid adapted cells [17]. 

**Figure 2:**
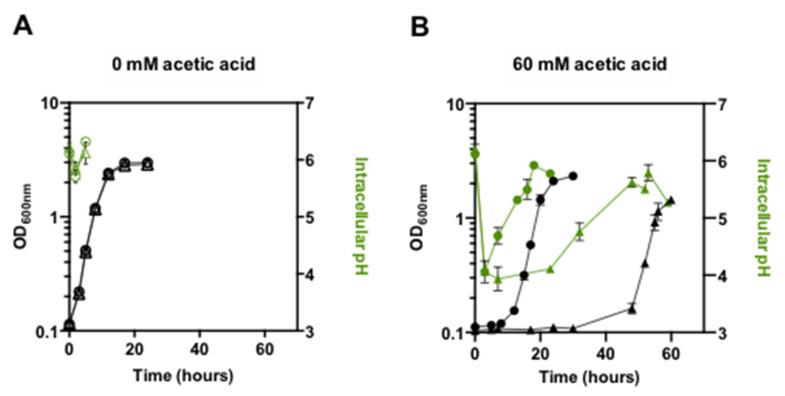
Alteration of the average intracellular pH (pHi) of populations of *S. cerevisiae* BY4741 (circles) and this strain with the *PDR18* gene deleted (triangles) during cultivation in the absence (**A**) or presence (**B**) of acetic acid stress, as described in Figure 1, legend. Intracellular pH was assessed by fitting the ratios of fluorescence in the equations obtained from the calibration curves for parental and *pdr18*Δ strains, which converts the fluorescence ratio to pHi values, as described in M&M. Values are the means of at least two independent experiments and error bars indicate standard deviations.

During exponential growth in unstressed conditions, the intracellular pH (pHi), assessed based on flow cytometry using the ratiometric pH indicator SNARF-4F 5-(and-6)-carboxylic acid, acetoxymethyl ester, and acetate, exhibits similar values (6.05 ± 0.3 and 6.06 ± 0.2) for both the parental and *pdr18*Δ populations, respectively (Figure 2A). However, when the cultivation medium was supplemented with the abovementioned sub-lethal concentration of acetic acid, the average pHi decreased significantly for both strains during the first 3 h of cultivation (Figure 2B). After 7 h of cultivation, the average pHi of the parental strain cell population recovered, accompanying the recovery and exponential growth of the weak acid-adapted cell population, and reached values close to the initial pHi. The deletion of *PDR18* leads to the extension of the duration of the lag phase and to lower pHi values. The recovery of pHi to more physiological values followed the resumption of exponential growth of an adapted population (Figure 2B). The average pHi attained for both populations at the late exponential growth phase was below the initial pHi registered for the cells of the inocula (5.92 ± 0.03 and 5.79 ± 1.4, *p* = 0.0002 and 0.008 for the parental and *pdr18*Δ strains, respectively; one-way-ANOVA; Figure 2B), with slightly lower values in the *pdr18*Δ deletion mutant cells. As the stationary phase progressed, the average pHi of both populations decreased, likely due to plasma membrane proton gradient dissipation as the result of ATP depletion [43,55,56].

### 3.2. Expression of PDR18 Is Required for Maximum Resistance of Yeast Cells to Lyticase Activity Induced under Acetic Acid Stress

**Figure 3:**
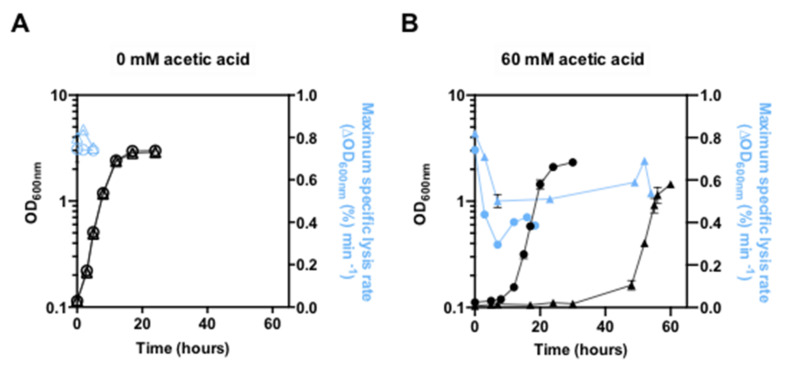
Yeast cell resistance to lyticase activity during cultivation in the absence or presence of acetic acid stress. Time-course analysis of cell susceptibility to lyticase, associated with the maximum specific lysis rate of the parental strain, *S. cerevisiae* BY4741 (circles), and this strain with the *PDR18* gene deleted (triangles). Cells were harvested from selected time-points during cultivation, in the absence (**A**) or presence (**B**) of acetic acid. The maximum specific lysis rate is defined as the absolute value of the slope of the straight line that best fits the semi-logarithmic plot of the linear part of the lysis curve and was determined based on the decrease of the OD_600nm_ of cell suspensions (in %) following the addition of lyticase, as described in M&M. Results from these lysis experiments are shown in Supplementary Figure S2. Values are means from at least two independent replicates of the lysis experiments, and bars represent standard deviation.

The alterations occurring in yeast cell wall architecture during cultivation of the BY4741 strain with the *PDR18* gene deleted, in the presence or absence of 60 mM acetic acid at pH 4.0, the same conditions used above and replicated for all the experiments described in this study, were monitored based on cell wall susceptibility to lyticase activity and compared with values of the parental strain (Figure 3). The maximum specific lysis rate at each time-point during cultivation was determined as the slope of the trend line that best fits the semi-logarithmic plot linear part of the decrease of the OD_600nm_ of the cell suspension following the addition of lyticase (Supplementary Figure S2). In the absence of acetic acid stress, the maximum specific lysis rate of the parental and the *pdr18*Δ strains’ populations exhibit similar values during exponential growth (Figure 3A). When parental strain cells were introduced in the same medium supplemented with acetic acid, a marked reduction of the lysis rate was observed reaching minimum values at 3 h of acetic acid-induced latency (Figure 3B). The reduction of the lysis rate for the *pdr18*Δ cell population, during the extended latency phase under acetic acid, was less marked than observed for the parental strain, and those reduced values were maintained throughout the duration of the more extended lag phase (Figure 3B). In the adapted populations of the parental or *pdr18*Δ strains exponentially growing under acetic acid stress, the lysis rate values moderately increased when compared to those registered during latency. However, these values were quite below those registered for unstressed cells and were lower for the parental strain compared with the *pdr18*Δ mutant (Figure 3B). In summary, results indicate that *PDR18* expression is required for the induction of maximum levels of cell wall resistance to lyticase activity during adaptation and growth under acetic acid stress.

### 3.3. The Stiffness of pdr18Δ Cell Wall Increases during Acetic-Acid-Induced Latency

**Figure 4:**
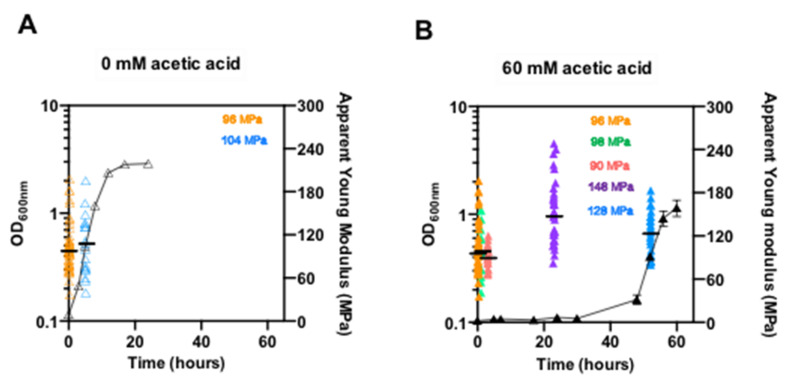
Apparent Young’s modulus of *S. cerevisiae* BY4741 with the *PDR18* gene deleted, cultivated in the absence or presence of acetic acid stress. Time-course analysis of the cell surface elasticity, which reflects cell wall stiffness, represented as the apparent Young’s modulus determined by atomic force microscopy, for *pdr18*Δ cells cultivated in the absence (**A**) or presence (**B**) of acetic acid. Each result in the graph corresponds to the median value of about 100 curves over a single cell. For each condition, at least 13 cells were analyzed from at least 3 independent experiments.

The nanomechanical properties of the cell surface of *pdr18*Δ cells during adaptation to acetic acid stress were examined by Atomic Force Microscopy (AFM) to assess the Young’s modulus, which reflects cell wall stiffness (Figure 4). Each experimental result corresponds to the median value of about 100 curves over a single cell, and the dispersion of the results for each cultivation time reflects the expected heterogeneity [57] of the yeast populations examined. As previously described for the parental strain [20], the Young’s modulus of *pdr18*Δ cells was not found to suffer significant alterations during exponential growth in unstressed conditions (Figure 4A). However, when the medium was supplemented with acetic acid using the above-described conditions, the Young’s modulus value for *pdr18*Δ cells increased during latency, reaching a maximum value of 148 MPa at 23 h of cultivation, when compared to the Young’s modulus value of 96 MPa of the initial time-point (*p* < 0.00001; one-tailed Mann–Whitney U test; Figure 4A,B). Moreover, the Young’s modulus of exponentially growing *pdr18*Δ cells, adapted to acetic acid (128 MPa), was found to be significantly higher than the Young’s modulus of the corresponding unstressed cell population (104 MPa; *p* = 0.04; one-tailed Mann–Whitney U test; Figure 4). Compared with the stiffness values published previously for parental strain cells [20], cultivated under exactly the same stress conditions and exhibiting the same growth curves and other parameters (Figure 1, Figure 2 and Figure 3), the values of cell stiffness obtained for the *pdr18*Δ strain under acetic acid stress were not statistically different from those reported for the parental cells; moreover, the profiles of variation of cell stiffness during the adaptation phase were similar (Figures 2b and 4 from reference [20]). 

### 3.4. Transcriptional Profiles of Cell Wall Biosynthetic Genes in pdr18Δ Cells’ Response to Acetic Acid Stress

The levels of transcription from several genes related to cell wall biosynthesis were assessed by quantitative real-time Reverse Transcription–PCR (qRT–PCR) during cultivation of the *pdr18*Δ strain in the absence or presence of acetic acid under the above-described conditions. The selected genes, the description of the function of the encoded proteins, and the corresponding bibliographic references are provided in Table 1.

**Figure 5:**
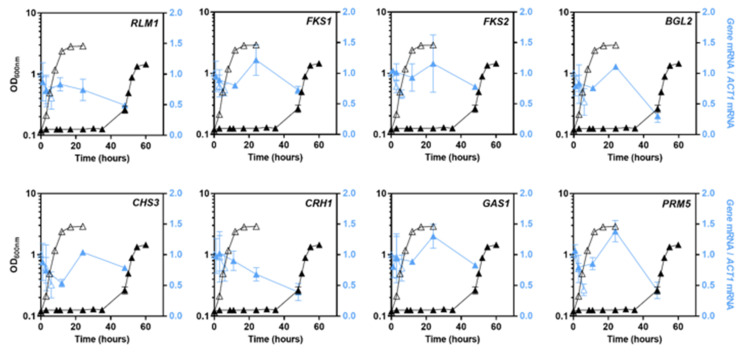
Levels of mRNA from cell wall biosynthesis-related genes during cultivation of *S. cerevisiae* BY4741 with the *PDR18* gene deleted and in the absence or presence of acetic acid. Time-course of the mRNA levels from *RLM1*, *FKS1*, *FKS2*, *BGL2*, *CHS3*, *CRH1*, *GAS1,* and *PRM5* genes during cultivation of *pdr18*Δ in the absence (open symbols) or presence (filled symbols) of acetic acid. The transcriptional levels (in blue) from the indicated genes were assessed by qRT–PCR, using *ACT1* as the internal control. The value obtained for each target gene, at the initial time-point of parental strain cell cultivation under unstressed conditions, was set as 1. Results are means of at least three biological replicates, and error bars represent standard deviation.

During the cultivation of the parental strain BY4741 in unstressed conditions (MM4, pH 4.0) the levels of transcription from all the selected genes, *RLM1*, *FKS1*, *FKS2*, *BGL2*, *CHS3*, *CRH1*, *GAS1,* and *PRM5,* were found not to vary significantly [20]. The same was observed herein for the *pdr18*Δ strain under the same cultivation conditions (Figure 5).

The alteration of the transcription levels from the selected genes in the parental strain *S. cerevisiae* BY4741, under the same acetic acid stressing conditions used in this work, was previously described [20]. While the mRNA levels from *RLM1*, *FKS2*, *BGL2*, *CHS3*, *CRH1,* and *PRM5* decreased during growth latency in the parental strain, mRNA levels from *FKS1* maintained similar values, and mRNA levels from *GAS1* increased moderately throughout cultivation [20]. In the present study, a different transcriptional profile was found for those genes in the strain with the *PDR18* gene deleted. Although a similar pattern of the time-course transcription levels was found herein for all genes, except for *RLM1* and *CRH1*, during the first 12 h of the latency phase, corresponding to a slight decrease of the mRNA levels during the first hours of response to sudden acetic acid stress, these changes were not considered statistically significant. At 24 h of cultivation, when the cell population was close to resuming growth, the mRNA levels from *FKS1*, *FKS2*, *BGL2*, *CHS3*, *GAS1,* and *PRM5* increased by 40%, 30%, 37%, 107%, 37%, and 73%, respectively, compared with the 12 h time-point values (Figure 5). The observed increase was statistically significant for *FKS1*, *BGL2, CHS3*, *GAS1*, and *PRM5* (*p* < 0.05; one-way ANOVA), with coding for a β-1-3-glucan synthase, an endo-beta-1,3-glucanase, a chitin synthase, a β-1,3-glucanosyltransferase, and a protein of unknown function considered to be a hallmark of CWI pathway activation [60], respectively (Figure 5). When cells resumed growth, the mRNA levels from these genes did not maintain the peak values attained by the end of the adaptation phase to acetic-acid-induced stress. In the case of the *RLM1* gene, coding for a transcription factor responsible for the transcriptional activation of the CWI pathway, and in the case of the *CRH1* gene, coding for a chitin transglycosylase, the referred expression pattern was not observed. In fact, the mRNA levels from these genes decreased throughout the time-points tested, down to 50 and 60% compared to the initial time-point, respectively (Figure 5).

### 3.5. The Content of the Major Cell Wall Polysaccharides Increases in pdr18Δ Cells during Acetic Acid-Induced Latency

**Figure 6:**
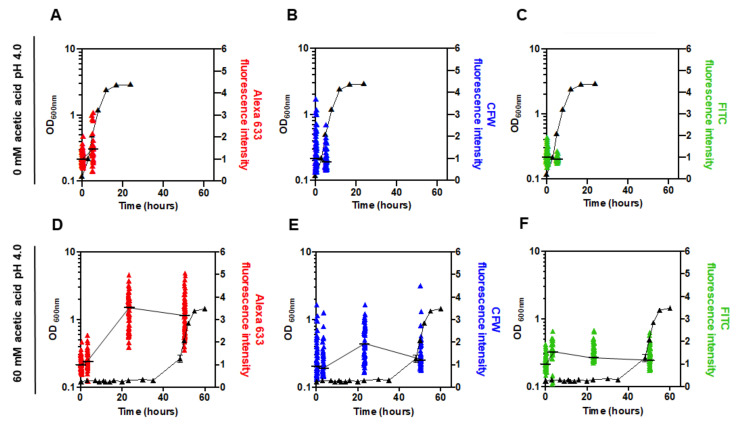
Content of cell wall polysaccharides in the absence and presence of acetic acid, in *S. cerevisiae* BY4741 with the *PDR18* gene deleted, measured by fluorescence microscopy. The estimated content of cell wall polysaccharides during cultivation of *pdr18*Δ cells in the absence (**A**–**C**) or presence (**D**–**F**) of acetic acid is shown. The cell wall components β-glucans (red), chitin (blue), and mannans (green) were stained with Fc-Dectin 1 conjugated with Alexa Fluor 633, Calcofluor White, and Concanavalin A conjugated with Fluorescein (FITC), respectively. Quantification of the fluorescence intensity was performed with Leica TCS SP5 (Leica Microsystems CMS GmbH, Manheim, Germany) inverted confocal microscope (DMI600). Each experimental result corresponds to the median fluorescence intensity value (indicated by a black dash) from at least 33 cells from two independent cultivation experiments, and the dispersion of the results for each cultivation time reflects the expected heterogeneity of the yeast populations examined. For each channel, the median of the intensity values obtained for *pdr18*Δ cells at the initial time-point (0 h) was set as 1, and the remaining values are relative to this reference value.

The evolution of the content of cell wall polysaccharides during adaptation and growth in the presence of acetic acid of the more acetic acid susceptible *pdr18*Δ cell population was assessed, as before for the parental strain population [20], using fluorescence microscopy. The cell wall polysaccharides β-glucans, chitin, and mannans were stained with Alexa Fluor 633 (AF633), Calcofluor White (CFW), and Concanavalin A conjugated with Fluorescein (FITC), respectively. The fluorescence intensity median values obtained for the individual *pdr18*Δ cell measurements during cultivation in the absence or presence of acetic acid are shown in Figure 6, whereas illustrative images acquired by fluorescence microscopy are shown in Supplementary Figure S3. Each experimental result corresponds to the median value from at least 33 cells from two independent cultivation experiments, and the dispersion of the results for each cultivation time reflects the expected heterogeneity [57] of the yeast populations examined.

No significant alteration was registered in the content of the cell wall polysaccharides when the parental cells of the *S. cerevisiae* BY4741 strain [20] or cells with the *PDR18* gene deleted were cultivated in the absence of acetic acid stress (Figure 6A–C). The study of the alterations occurring in cell wall polysaccharides during adaptation of parental strain cells to the same acetic acid stressing conditions used in the present study was previously published and indicates that the level of β-glucans increases during adaptation and in adapted cells compared to unstressed cells [20]. Under the same stressing conditions, in the case of the more susceptible *pdr18*Δ cell population examined in this study, a statistically significant increase in the content of β-glucans, well above the increase observed before for parental cells [20], also occurred during the adaptation phase. A peak for β-glucan content was reached after 23 h of cultivation (3.6-fold higher than the initial time-point; *p* < 0.00001; one-tailed Mann–Whitney U test), when the population was about to resume exponential growth (Figure 6D). These high values were maintained during exponential growth, and values were 2.2-fold higher when compared to the corresponding unstressed cells (*p* < 0.00001; one-tailed Mann–Whitney U test; Figure 6A,D). 

The chitin content, associated with the increase of CFW fluorescence intensity, also peaked at 23 h of cultivation (1.9-fold higher than the initial time-point; *p* < 0.00001; one-tailed Mann–Whitney U test) and maintained values 1.5-fold higher than those of unstressed cells (*p* < 0.00001; one-tailed Mann–Whitney U test) during exponential growth (Figure 6B,E). The chitin content variation profile was similar to the profile of variation of the β-glucans (Figure 6D,E). Since CFW can also stain β-glucans to a smaller extent [64], it is not possible to be sure if there is indeed a specific increase of the chitin concentration or if this increase just reflects the increased content of β-glucans (Figure 6E). 

A statistically significant but smaller increase in the content of mannans, quantified by the fluorescence of Concanavalin A-fluorescein (FITC) (1.6-fold higher than the initial time-point; *p* < 0.00001; one-tailed Mann–Whitney U test) also occurred. However, the peak value was reached earlier in the latency phase, after 3 h of cultivation in the presence of acetic acid (Figure 6F). The cell wall content of mannans in acetic acid stressed cells was also slightly higher than the level in cells of the inoculum, which were grown in the absence of acid stress (1.3-fold higher; *p* < 0.00001; one-tailed Mann–Whitney U test; Figure 6C,F). In the previous work studying the parental strain cell wall composition under the same stressing conditions, the increase in the content of mannans in response to acetic acid stress was smaller and not statistically significant, although such an increase was already apparent [20].

## 4. Discussion

Among the relevant mechanisms proposed to underlie yeast adaptation to stress imposed by a sub-lethal concentration of acetic acid is the alteration of the composition, organization, and properties of the cell envelope [2,17,18,19,20]. The present work reinforces the concept of the major role played by the cell wall in the global response and tolerance to acetic acid stress in yeast. Moreover, it provides evidence for the crosstalk between the content of the plasma membrane in ergosterol, with this depending on the activity of the plasma membrane ABC transporter Pdr18 and the biophysical properties of the cell wall under acetic acid stress.

Cell wall remodeling is considered an essential response to limit the futile cycle associated with the re-entry of the toxic form of acetic acid, after the active expulsion of the counter-ion acetate, accumulated in the cell interior, due to a cytosolic pH well above the pKa of this weak acid [2,20]. Acetate extrusion was proposed to be mediated by drug-H^+^ antiporters (Tpo2, Tpo3, Aqr1) [40,41,42], while the re-entry of the lipophilic acid form is possible by passive diffusion through the plasma membrane lipid bilayer [2,65]. The more active acetate and proton effluxes during adaptation to acetic acid are energy-dependent mechanisms [65], leading to the decrease of the ATP pool under acetic acid stress [65]. 

In the present work, it was demonstrated that the expression of *PDR18* contributes to counteract the rapid and marked decrease of cellular ergosterol content, also registered during the acetic-acid-induced adaptation phase. The extended period of growth latency observed for the mutant with the *PDR18* gene deleted was correlated with the lower levels of ergosterol present in these cells and the longer duration required for the cell population pHi to recover from the minimum pHi value of approximately 4.0 to more physiological levels and resume exponential growth. Therefore, the expression of *PDR18* was demonstrated to allow the maintenance of physiological levels of ergosterol, required to overcome the severe deleterious effects of acetic-acid-induced stress at the plasma membrane level, such as the coordinated decrease of the plasma membrane order, increase of the non-specific plasma membrane permeability, and decrease of the transmembrane electrochemical potential [17]. The more extended period of latency during which *pdr18*Δ cells maintain a low pHi is consistent with the reported profile of increased acetic-acid-induced permeability in this mutant cell population [17].

Impaired mannosylinositol phosphorylceramide (MIPC) biosynthesis was found to result in increased yeast cell susceptibility to zymolyase [66], an enzyme mixture that, similar to lyticase, has predominantly β-glucanase activity. This increased susceptibility to zymolyase was partially relieved by the overexpression of *ERG9*, encoding an enzyme of the ergosterol biosynthetic pathway [66]. Increased sensitivity to zymolyase was also reported for the *erg6*Δ mutant [67], highlighting the importance of ergosterol biosynthesis in cell wall integrity. In this study, Pdr18 was found to be required for maximum cell resistance to lyticase activity under adaptation and growth under acetic acid stress, reinforcing the idea that plasma membrane lipid composition and homeostasis influence cell wall integrity under stress. 

The coordinated increase of yeast cell wall stiffness and content of the major cell wall polysaccharides during adaptation to acetic-acid-induced stress was observed in cells lacking the *PDR18* gene, as reported before for the parental strain [20]. Although the most dramatic increase was observed for β-glucans, apparently, the content of the cell wall in chitin and mannans also increases during the acetic-acid-induced lag phase and in adapted cells. Since mannoproteins have an important role in controlling the porosity of the cell wall [68,69,70,71], the increased content of mannans in the *pdr18*Δ cell wall may contribute to restricting the diffusional entry of acetic acid in the cell. Some glycosyl-phosphatidylinositol (GPI) proteins, which are mannoproteins anchored via a C-terminus GPI anchor remnant in the plasma membrane [37,72,73], are involved in cell wall remodeling and determinants of acetic acid tolerance in *S. cerevisiae* [9]. This is the case for the GPI-protein encoded by *GAS1*, found to be slightly up-regulated during the yeast response to acetic acid ([20] and this work). Interestingly, plasma membrane lipid composition, in particular concerning the content in ergosterol and complex sphingolipids, determines the proper raft association of Gas1 and sorting of the plasma membrane [74], ultimately influencing its activity. Interestingly, a recent QTL mapping to uncover the genetic basis that confers to the bioethanol industrial strain Pedra-2 (PE-2) tolerance during growth at low pH, identified a non-synonymous mutation (A631G) in *GAS1*, prevalent in wild-type isolates and absent in laboratory strains [75]. The chitin content, assessed by the quantification of calcofluor white fluorescence intensity, also increased during acetic-acid-induced latency, with a time-course pattern similar to the β-glucan profile. Since this *fluorophore* also has an affinity for β-glucans, although to a less extent than for chitin [64], chitin quantification may include some contribution from the increase of cell wall β-glucans. However, given that our results also show that under acetic acid stress the transcription level from the *CHS3* gene, encoding the major chitin synthase, increased, as well as cell surface stiffness, which is dependent on the cross-linking between chitin and β-glucans [24,25], it is likely that the fluorescence values truly reflect the increase of chitin content in *pdr18*Δ cells cultivated under acetic acid stress. 

Cell-to-cell heterogeneity within a population is known to be an important factor contributing to acetic acid tolerance in *S. cerevisiae* [57,76,77]. Cells devoid of *PDR18* were previously found to exhibit a higher cell-to-cell heterogeneity concerning plasma membrane order when exponentially growing in the presence of acetic acid [17]. In the present study, the cell-to-cell analysis of cell stiffness and polysaccharide content also suggest a higher cell-to-cell heterogeneity for the *pdr18*Δ cell population under acetic acid stress as compared to the parental strain [20]. 

The transcription levels from the *FKS1, BGL2, GAS1, CHS3,* and *PRM5* genes were found to significantly increase during the adaptation phase to acetic acid before growth resumption under stress. The *FKS1, BGL2*, *GAS1,* and *CHS3* genes code for a β-1,3-synthase [59], an endo-β-1,3 glucanase involved in β-1,3 branching [62], β-1,3-glucanosyltransferase, which leads to elongation of (1→3)-β-D-glucan chains [62], and a chitin synthase [58]. This coordinated gene up-regulation apparently correlates with the higher content of β-glucans and chitin, and the increased stiffness registered for the *pdr18*Δ cell population at the same phase of growth under acetic acid stress, compared with unstressed cells. Consistent with our results and interpretation, it was reported that either yeast mutations or exposure to fluconazole, leading to an ergosterol decrease, results in increased cell wall chitin content [78,79]. The very rapid and dramatic reduction in the ergosterol content occurring during the first hour of cultivation in the presence of the acid for the parental and the *pdr18*Δ populations is herein described for the first time. This reduction can be related with acetic-acid-induced intracellular acidification, which affects biosynthetic enzyme activity. Since ergosterol biosynthesis is an energetically expensive process [80], this process is affected in cells requiring energy for ATP-dependent mechanisms of adaption to acetic acid stress (e.g., recovery of pHi, acetate efflux). Results from studies on the reprogramming of genome-wide expression during acetic-acid-induced stress include the increased transcription from genes encoding the ergosterol biosynthetic pathway, necessary to counteract acetic-acid-induced reduction of the ergosterol content [9,10,17]. Although *pdr18*Δ cells exhibit a lower cellular ergosterol content when compared to the parental strain, during the long-term response to acetic acid stress of the mutant cells, the increase of the ergosterol content was also observed, with this response being coordinated in time with more favorable cell wall biophysical alterations. It is hypothesized that yeast cells lacking Pdr18, and therefore containing lower ergosterol levels and a less ordered and more permeable plasma membrane, respond more intensively to the same acetic acid concentration to remodel cell wall composition and organization.

**Figure 7:**
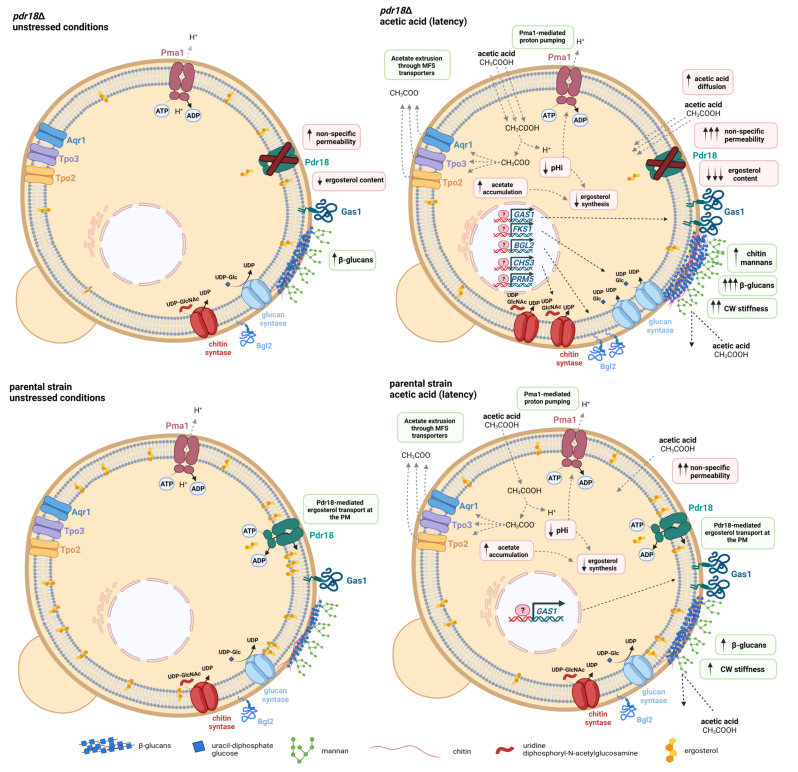
Schematic model for the adaptive response to acetic-acid-induced stress in the parental and *pdr18*∆ strains, at the level of the cell envelope, suggested by the results of the present study. The adaptation mechanisms (described in green boxes) to counteract the deleterious effects of acetic acid stress (described in red boxes) are represented for both strains. The arrows in grey represent previous findings, and the black arrows represent the results obtained in this work.

Collectively, our results contribute to a better understanding of the time-course alterations occurring at the level of the cellular envelope during yeast adaptation to acetic acid stress, in particular of cells lacking Pdr18 (Figure 7). Compared to the parental strain, cells devoid of Pdr18 exhibit lower levels of ergosterol and a more extended latency phase, during which the low pHi is maintained. As observed for parental cells, adaptation to acetic acid stress in *pdr18*Δ cells requires cell wall remodeling, clearly detected in the late phase of growth latency. Such remodeling is characterized by increased contents of β-glucans, chitin, and mannans in the cell periphery, reflecting the observed up-regulation of transcription from genes involved in β-glucan and chitin synthesis and/or elongation. Altogether, these modifications contributed to an increase of cell stiffness when the *pdr18*Δ cell population was about to resume exponential growth, above the values registered for the parental cell population in the same conditions. However, despite the more intense response of *pdr18*Δ to acetic acid stress, the induced increase of *pdr18*Δ cell resistance to lyticase activity is below the values for parental cells, suggesting a role for yeast plasma membrane composition and order in cell wall architecture. The described adaptation profiles, involving all the cell envelope parameters examined in this study, correlate with the profile of recovery to more physiological pHi values and growth resumption under acetic acid stress. This study provides relevant new information on how plasma membrane homeostasis engineering can be used for the rational development of superior yeast strains more tolerant to acetic acid. The modulation of plasma membrane ergosterol content and of the expression of ABC transporters involved in lipid homeostasis is pointed out herein as a promising molecular target for the improvement of acetic acid. This is an essential trait to increase the economic sustainability of lignocellulosic biorefineries.

## Figures and Tables

**Table 1:** Genes selected for qRT–PCR, description of the function of the encoded proteins, and corresponding references.

Gene	Function of the Encoded Protein	Bibliographic References
*CHS3*	Major chitin synthase required for the synthesis of the majority of cell wall chitin	[58]
*FKS1*	β-1-3-glucan synthase	[59]
*FKS2*	Fks1 paralog, involved in β-1-3-glucan synthesis	
*RLM1*	Transcription factor responsible for the transcriptional activation of the majority of genes induced in response to cell wall stress through the CWI pathway	[60]
*GAS1*	β-1,3-glucanosyltransferase involved in cell wall remodeling-elongation of (1→3)-β-D-glucan chains and branching	[61,62]
*CRH1*	Chitin transglycosylase involved in the transfer of chitin to β-1-6 and β-1-3 glucans in the cell wall	[63]
*BGL2*	Endo-beta-1,3-glucanase involved cell wall remodeling necessary for branching of the β-1-3 glucans in the cell wall	[62]
*PRM5*	Pheromone-regulated protein and an Rlm1 target; a hallmark of CWI pathway activation	[60]

## Data Availability

Data is available in this article and as Supplementary Materials.

## Supplementary Materials

The following are available online at https://www.mdpi.com/article/10.3390/jof8020103/s1, Figure S1: Calibration curves of SNARF-4F-5-(and-6)-carboxylic acid, acetoxymethyl ester, acetate. The calibration curves for parental and *pdr18*Δ populations convert the fluorescence ratio to pHi values. They were obtained by plotting the fluorescence ratio of the different samples as a function of the pH of the buffer, with a defined pH, in which they were incubated. The equations obtained results from the fitting of a second-order polynomial function; Figure S2: Lyticase susceptibility assays using cells of the parental and *pdr18*Δ strains harvest during the growth curve in the absence or presence of acetic acid. The decrease of the OD_600nm_ during the incubation time of yeast cell suspensions (in %) following the addition of lyticase is plotted; Figure S3: Fluorescence microscopy images of fluorescence-stained cell wall β-glucans, chitin and mannans. Illustrative images of stained *pdr18*Δ cells were taken after 0 h (A) and 23 h (B) of exposure to acetic acid, this corresponding to the middle of the acetic acid-induced latency. Confocal microscopy was used to quantify β-glucans and mannans stained with Fc-Dectin 1-Alexa 633 and Concanavalin A-FITC, respectively, and 2-photon excitation microscopy was used to quantify chitin stained with Calcofluor White; Table S1: Primers used for qRT-PCR analysis.
