# Supplementary material for: Crosstalk between Yeast Cell Plasma Membrane Ergosterol Content and Cell Wall Stiffness under Acetic Acid Stress Involving Pdr18
Source: J Fungi (Basel). 2022 Jan 21;8(2):103. doi: 10.3390/jof8020103 (PMC8880318; doi:10.3390/jof8020103)
Supplement: Supplementary file 1 [file jof-08-00103-s001.zip › jof-1554510-supplementary.pdf]

Supplementary Material

Crosstalk between yeast cell plasma membrane ergosterol content and cell wall stiffness under acetic acid stress involving Pdr18

Ricardo A Ribeiro, Cláudia P. Godinho, Miguel V. Vitorino, Tiago T. Robalo, Fábio Fernandes, Mário S. Rodrigues, Isabel Sá-Correia*

***Corresponding author:** isacorreia@tecnico.ulisboa.pt

Figure S1 – Calibration curves of SNARF-4F-5-(and-6)-carboxylic acid, acetoxymethyl ester, acetate. The calibration curves for parental and *pdr18*Δ populations convert the fluorescence ratio to pHi values. They were obtained by plotting the fluorescence ratio of the different samples as a function of the pH of the buffer, with a defined pH, in which they were incubated. The equations obtained result from the fitting of a second-order polynomial function.

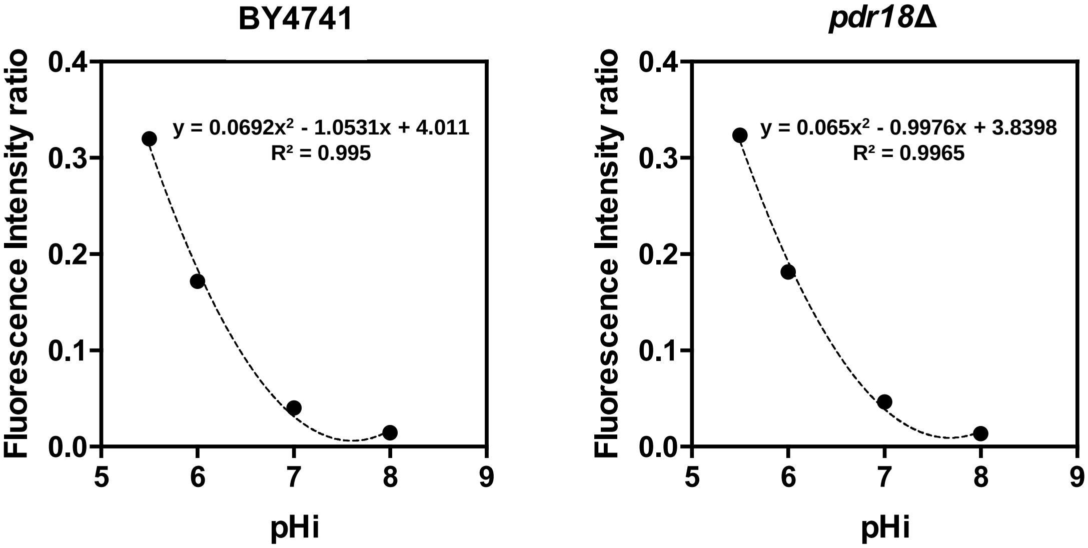

Figure S2 – Lyticase susceptibility assays using cells of the parental and *pdr18*Δ strains harvest during the growth curve in the absence or presence of acetic acid. The decrease of the OD_{600nm} during the incubation time of yeast cell suspensions (in %) following the addition of lyticase is plotted.

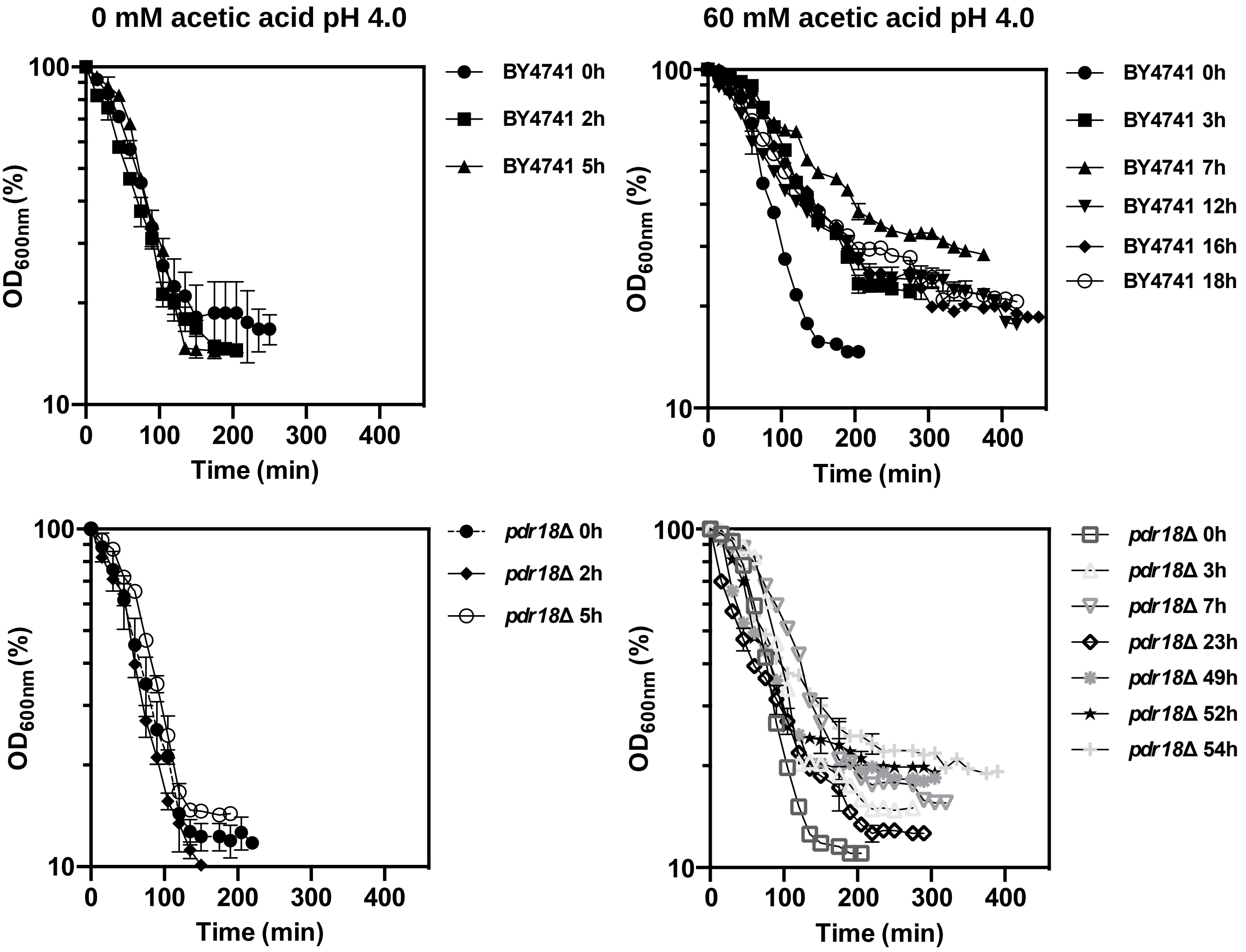

Figure S3 – Fluorescence microscopy images of fluorescence-stained cell wall β-glucans, chitin and mannans. Illustrative images of stained *pdr18*Δ cells were taken after 0 hours (A) and 23 hours (B) of exposure to acetic acid, this corresponding to the middle of the acetic acid-induced latency. Confocal microscopy was used to quantify β-glucans and mannans stained with Fc-Dectin 1-Alexa 633 and Concanavalin A-FITC, respectively, and 2-photon excitation microscopy was used to quantify chitin stained with Calcofluor White.

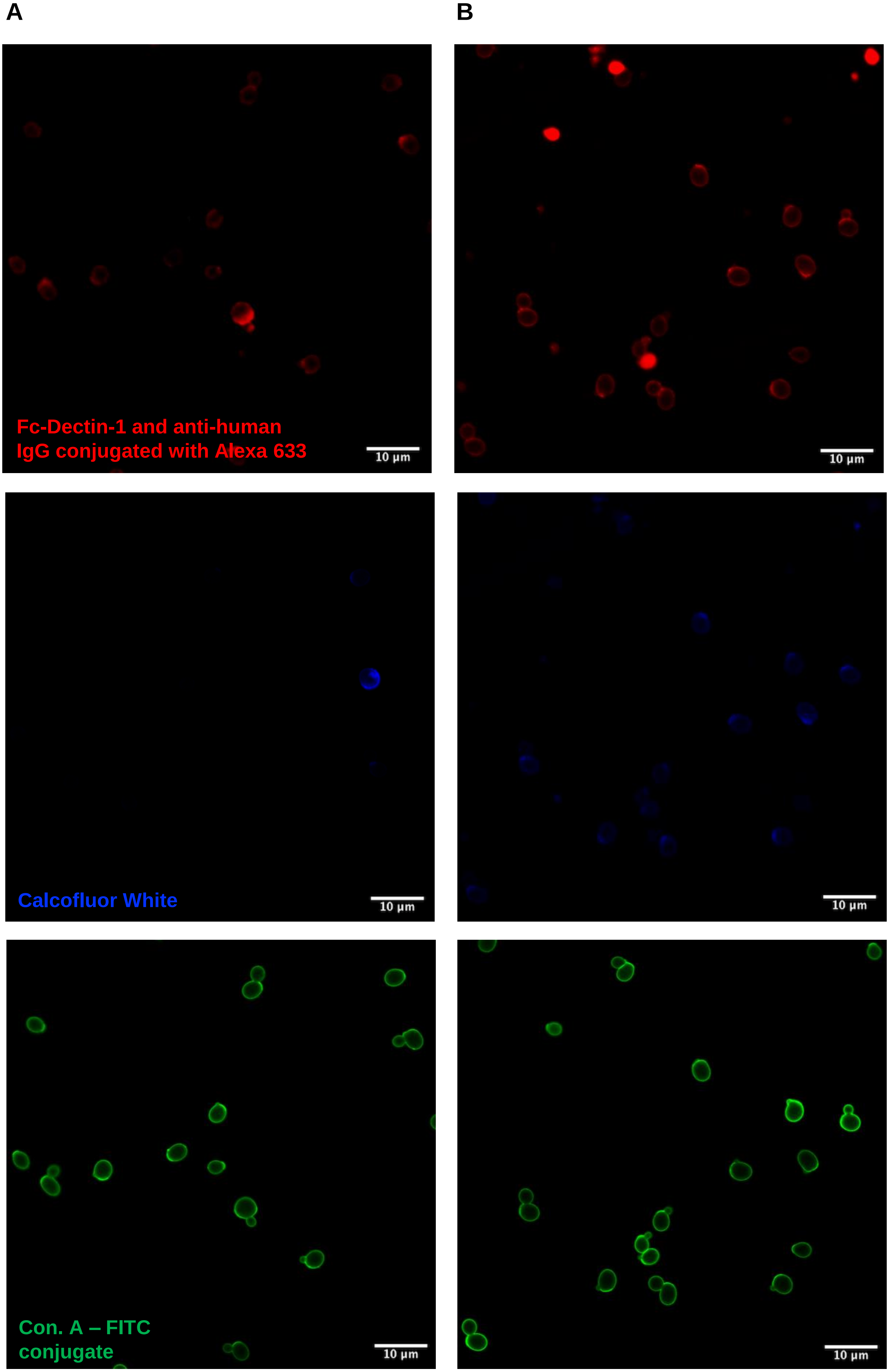

Table S1 - Primers used for qRT-PCR analysis.

Primer	Sequence (5'-3')
ACT1	fw: CTCCACCACTGCTGAAAGAGAA<br>rev: CCAAGGCGACGTAACATAGTTTT
CHS3	fw: TCACCTGGATGTTTTACCATCAAG<br>rev: CCACTCCGACGAGTTGCAT
FKS1	fw: CATGCTGCTCTGGTCCCTTATT<br>rev:CACCGTGGGCAATTCCA
FKS2	fw: GCTCATGTCGTTGGAGCAGTT<br>rev: CCAATGGCATTACGGAAAAGA
RLM1	fw: CTTTTTTCTGCAACACAGCCATA<br>rev: CGCCAGGAATATTCGATGGT
GAS1	fw: AACCGCTGCTGCTTTTTTTG<br>rev: CTTCAATCGCTGGAACATCGT
CRH1	fw: CGCGGCTGCCGAAAG<br>rev: GCA GTGCTAGAAGCTGCAGTTG
BGL2	fw: TTTTGTTATGGCTAACGCGTTCT<br>rev: GAGTAAGAGGCATTTTGCATGGT
PRM5	fw: TTTTCCACACAACATACCCAGTTT<br>rev: TCTTTGGCGGGATAATCCATA
